# Supplementary figures and images for: Neurotrophic factor Neuritin modulates T cell electrical and metabolic state for the balance of tolerance and immunity
Source: eLife. 2024 Nov 20;13:RP96812. doi: 10.7554/eLife.96812 (PMC11578584; doi:10.7554/eLife.96812)

NRN1  
15KD →

CD4 T cell sample

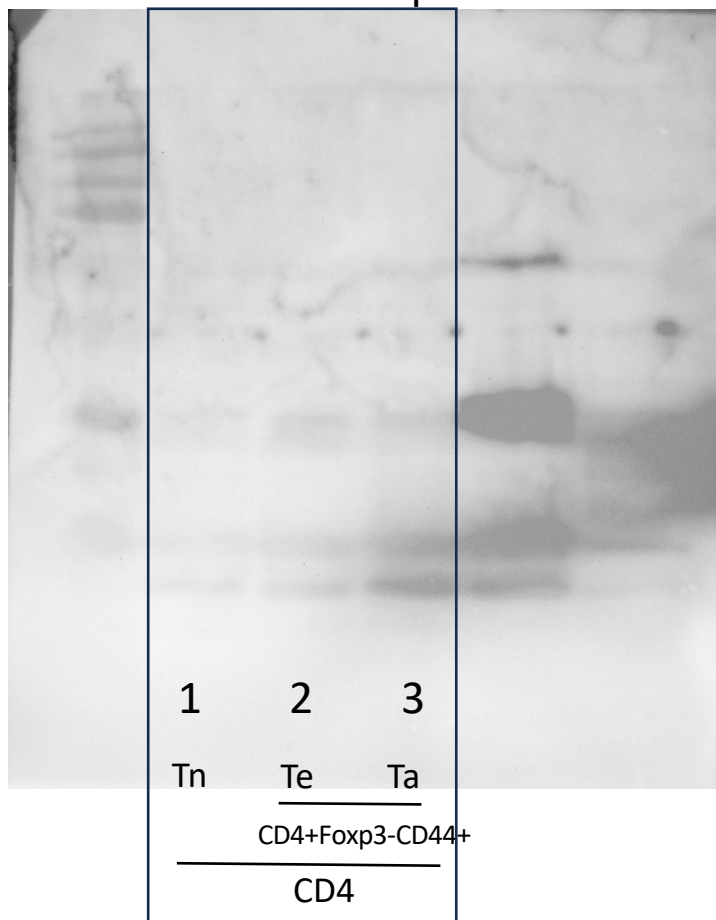

CD4 T cell sample

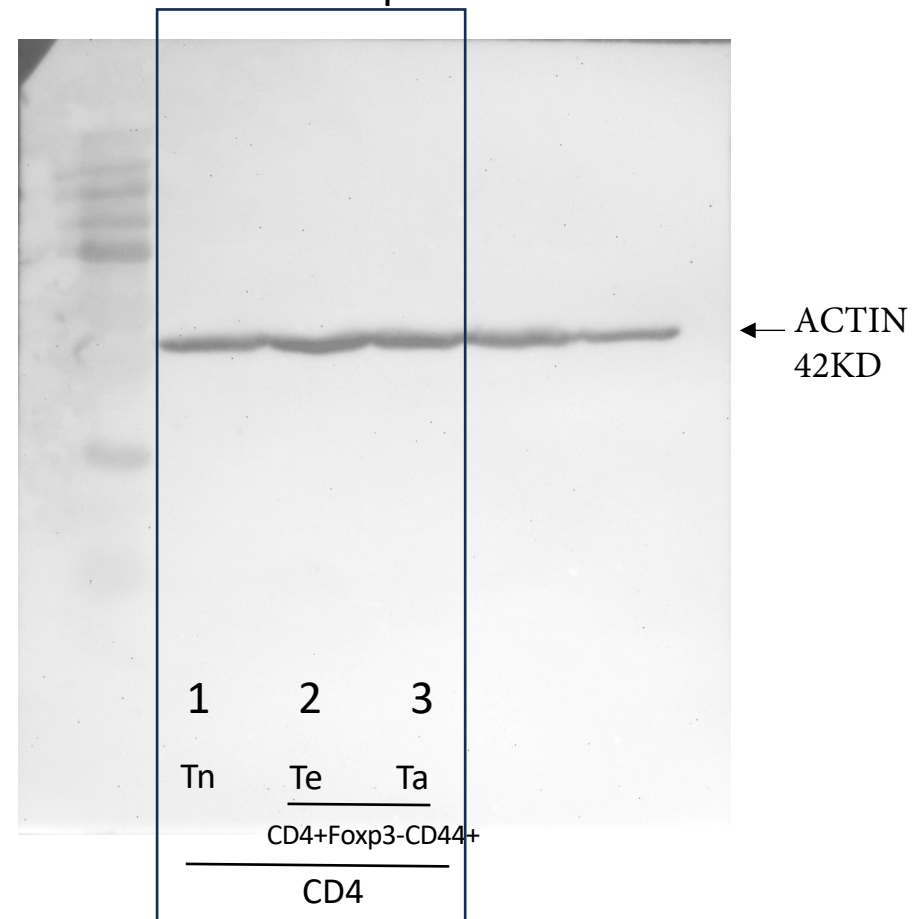

Supplement: Figure 1—source data 1. [file elife-96812-fig1-data1.zip › Figure 1-source data1/Figure 1-source data 1.pdf]

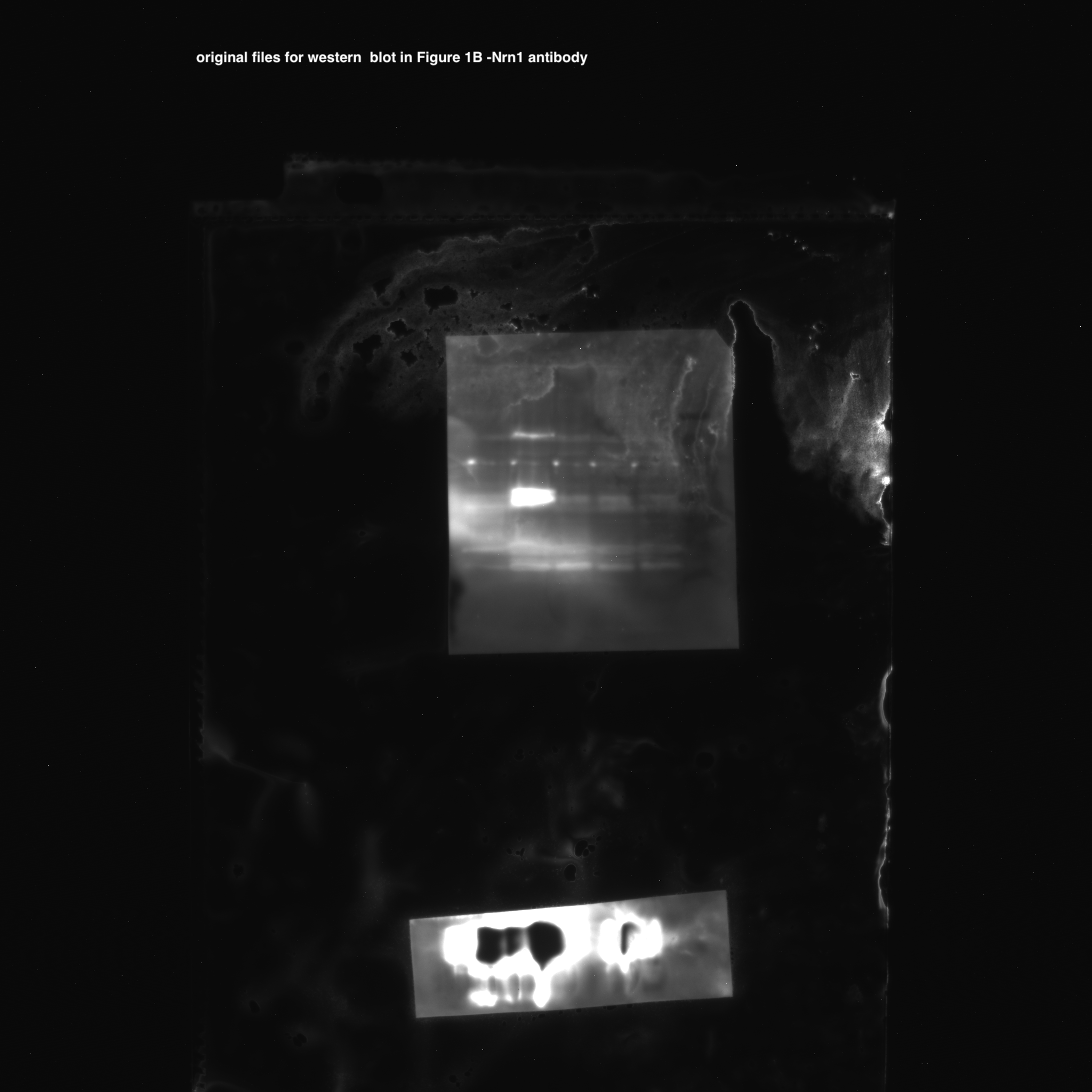

Supplement: Figure 1—source data 2. [file elife-96812-fig1-data2.zip › Figure 1-source data 2/Figure 1B Nrn1 antibody.tiff]

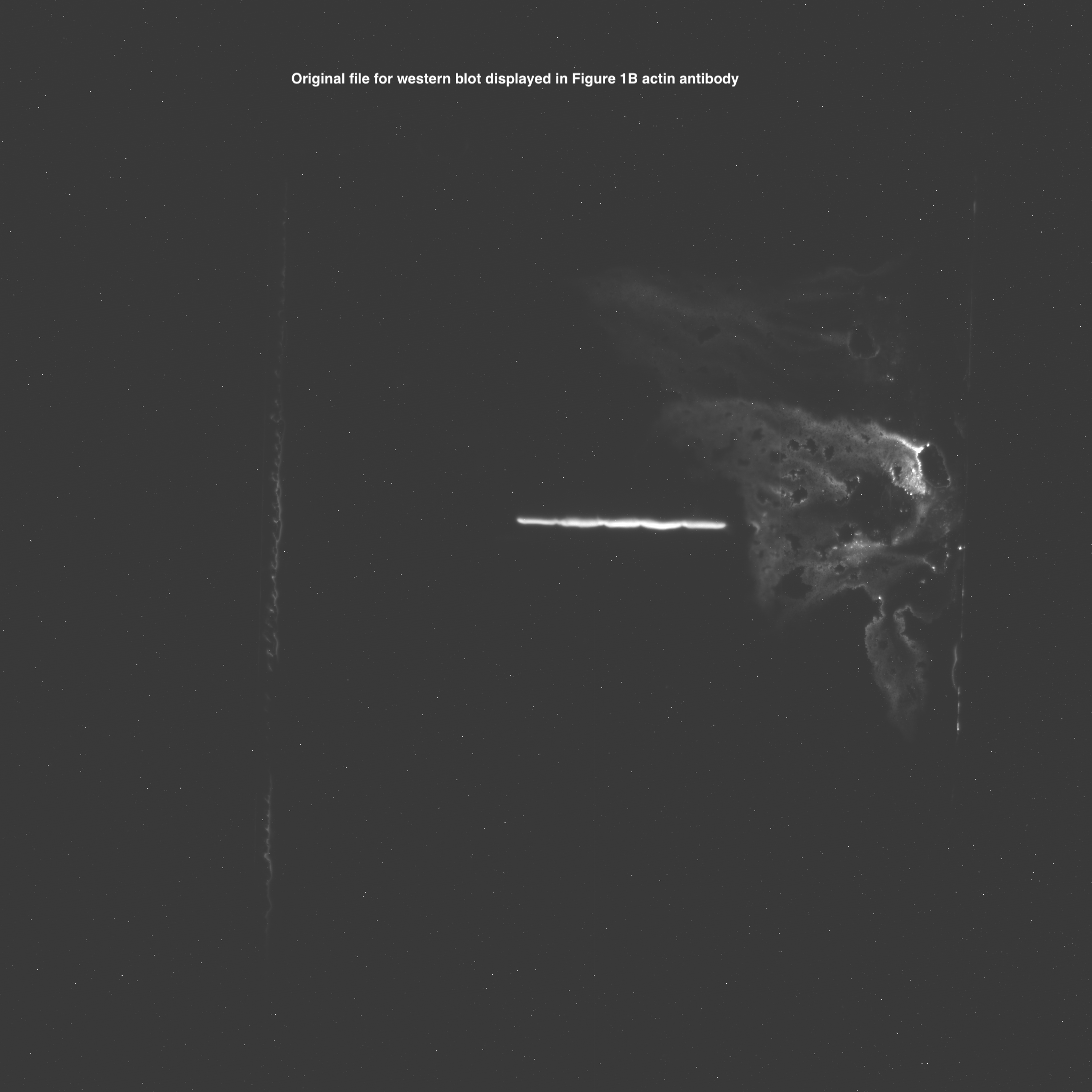

Supplement: Figure 1—source data 2. [file elife-96812-fig1-data2.zip › Figure 1-source data 2/Figure 1B actin antibody.tiff]

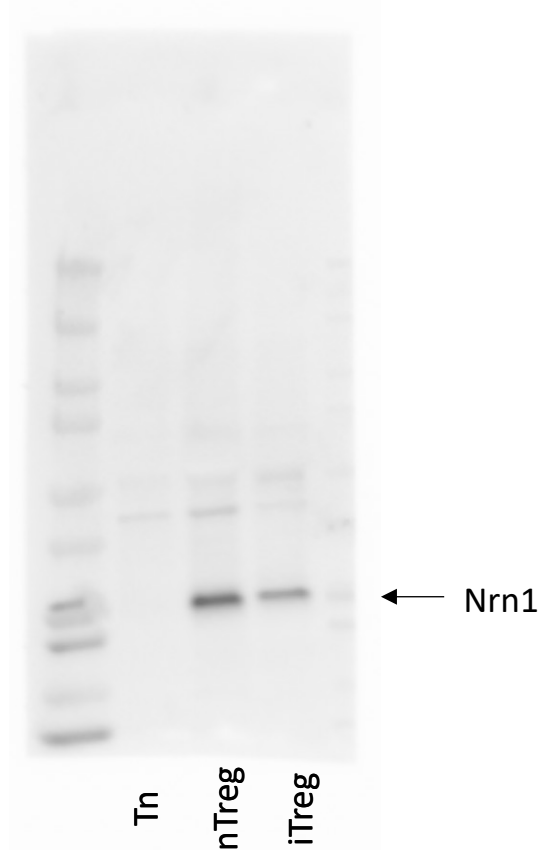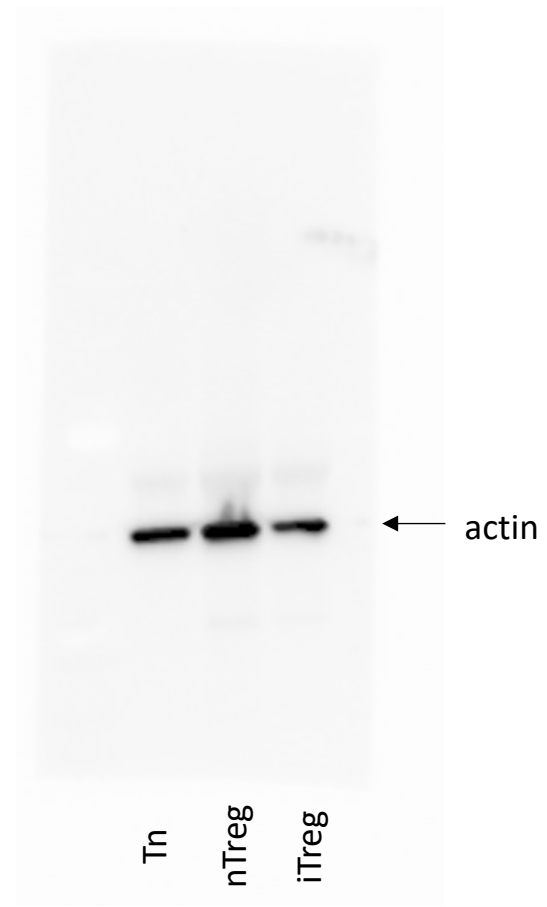

Supplement: Figure 1—source data 3. [file elife-96812-fig1-data3.zip › Figure 1-source data 3/Figure 1-source data 3.pdf]

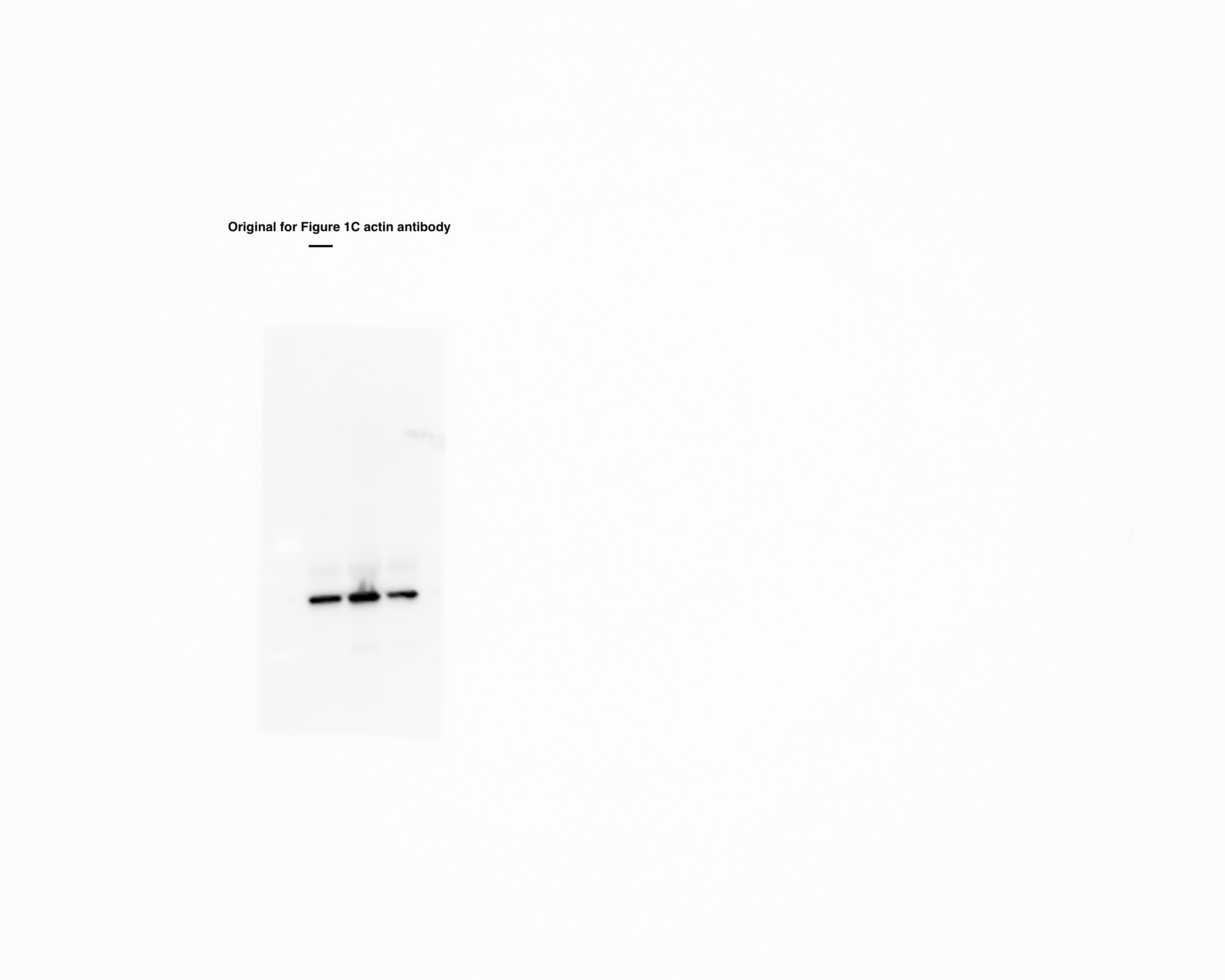

Supplement: Figure 1—source data 4. [file elife-96812-fig1-data4.zip › Figure 1-source data 4/Figure 1-source data 6.tiff]

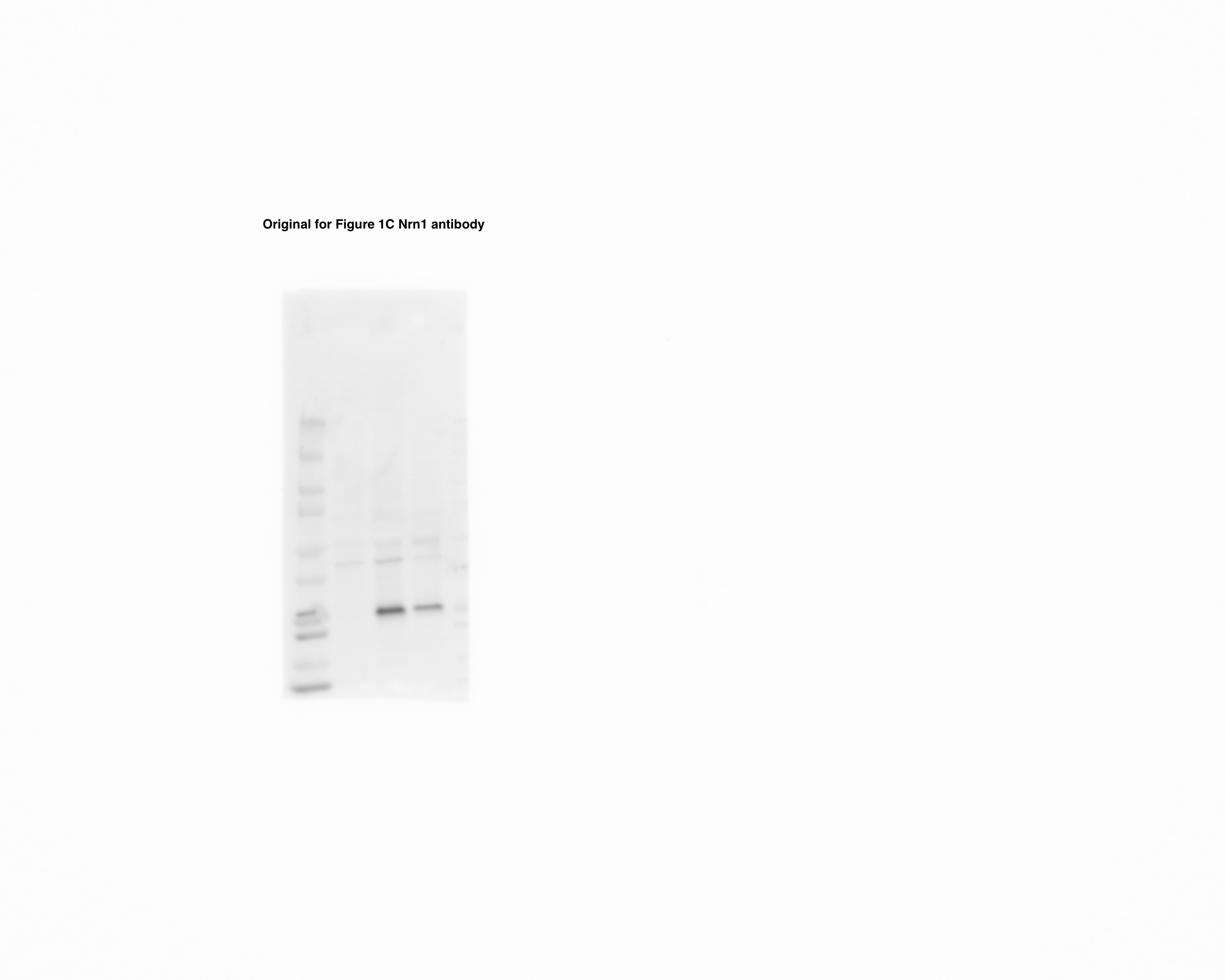

Supplement: Figure 1—source data 4. [file elife-96812-fig1-data4.zip › Figure 1-source data 4/Figure 1-source data 5.tiff]
