# Supplementary material for: Neurotrophic factor Neuritin modulates T cell electrical and metabolic state for the balance of tolerance and immunity
Source: eLife. 2024 Nov 20;13:RP96812. doi: 10.7554/eLife.96812 (PMC11578584; doi:10.7554/eLife.96812)
Supplement: MDAR checklist [file elife-96812-mdarchecklist1.pdf]

Westbrook Centre
Milton Road
Cambridge CB4 1YG, UK
P 01223 855340
W elifesciences.org
T @elife

Materials Design Analysis Reporting (MDAR) Checklist for Authors

The MDAR framework establishes a minimum set of requirements in transparent reporting mainly applicable to studies in the life sciences.

eLife asks authors to **provide detailed information within their article** to facilitate the interpretation and replication of their work. Authors can also upload supporting materials to comply with relevant reporting guidelines for health-related research (see EQUATOR Network), life science research (see the BioSharing Information Resource), or animal research (see the ARRIVE Guidelines and the STRANGE Framework; for details, see *eLife*'s Journal Policies). Where applicable, authors should refer to any relevant reporting standards materials in this form.

For all that apply, please note **where in the article** the information is provided. Please note that we also collect information about data availability and ethics in the submission form.

Materials:

Newly created materials	Indicate where provided: section/figure legend	N/A
Nrn1 antibody clone 1A10<br>Nrn1 antibody clone 1D6	Fig. 1 B-D, Fig.1-figure supplement 1C&D<br>Fig. 3-figure supplementary 2	

Antibodies	Indicate where provided: section/figure legend	N/A
All commertial antibody and clones are listed in the material methods section and in the key resources table.	Fig. 1H, Fig. 2, Fig. 3,Fig. 4, Fig. 5	N/A

DNA and RNA sequences	Indicate where provided: section/figure legend	N/A
N/A		N/A

Cell materials	Indicate where provided: section/figure legend	N/A
	N/A	
Primary culture from Nrn1-/- mice and C56/BL6 mice	Fig. 1, 2, 3, 4, 5	N/A

Experimental animals	Indicate where provided: section/figure legend	N/A
animal model listed in key resources table	key resources table	
		N/A

Plants and microbes	Indicate where provided: section/figure legend	N/A
N/A		N/A
		N/A

Human research participants	Indicate where provided: section/figure legend) or state if these demographics were not collected	N/A
N/A		N/A

Design:

Study protocol	Indicate where provided: section/figure legend	N/A
N/A		N/A

Laboratory protocol	Indicate where provided: section/figure legend	N/A
N/A		N/A

Experimental study design (statistics details) *		
For in vivo studies: State whether and how the following have been done	Indicate where provided: section/figure legend. If it could have been done, but was not, write "not done"	N/A
	Yes - see the Methods section entitled "Statistical analysis".	
	Yes - see the Methods section entitled "Statistical analysis".	
	Yes - see the Methods section entitled "Statistical analysis".	
	Yes - see the Methods section entitled "Statistical analysis".	

Sample definition and in-laboratory replication	Indicate where provided: section/figure legend	N/A
three to more times	Experiments were replicated at least 3 times. Sample sizes are shown in Tables and Figure legends, and represent biological replicates (individual cells or animals).	

Define whether data describe technical or biological replicates.	Data describe biological replicates (individual cells or animals).	

Ethics	Indicate where provided: section/submission form	N/A
		N/A
Studies involving experimental animals: State details of authority granting ethics approval (IRB or equivalent committee(s), animal protocole number M019M233	Research involving vertebrate animals was done at the Johns Hopkins University following protocols reviewed and approved by the Institutional Animal Care and Use Committee IACUC) - Animal Welfare Assurance Number M019M233. . The animals were housed in NIH-approved facilities and are observed daily by technicians. Unusual events are reported to the on call veterinarian, as well as to the investigator according to posted protocols. Other maintenance veterinary care was conducted according to NIH guidelines on the Use and Care of Animals. Facilities were inspected regularly according to NIH and AAALAC guidelines.	
		N/A

Dual Use Research of Concern (DURC)	Indicate where provided: section/submission form	N/A
		N/A

Analysis:

Attrition	Indicate where provided: section/figure legend	N/A
	All data generated or analyzed are included in the figures.	

Statistics	Indicate where provided: section/figure legend	N/A
Student t-test or ANOVA	See the Methods section entitled “Statistical analysis”.	

Data availability	Indicate where provided: section/submission form	N/A
For newly created and reused datasets, the manuscript includes a data availability statement that provides details for access (or notes restrictions on access).	All data associated with this study are present in the paper or the Supplementary Materials.	
		N/A

Code availability	Indicate where provided: section/figure legend	N/A
N/A		
		N/A

If reused code is publicly available provide accession number in repository OR DOI OR URL, OR citation.		N/A

Reporting:

The MDAR framework recommends adoption of discipline-specific guidelines, established and endorsed through community initiatives.

**Adherence to community standards**	**Indicate where provided: section/figure legend**	**N/A**
State if relevant guidelines (e.g., ICMJE, MIBBI, ARRIVE, STRANGE) have been followed, and whether a checklist (e.g., CONSORT, PRISMA, ARRIVE) is provided with the manuscript.		N/A

---

* We provide the following guidance regarding transparent reporting and statistics; we also refer authors to Ten common statistical mistakes to watch out for when writing or reviewing a manuscript.

Sample-size estimation

- You should state whether an appropriate sample size was computed when the study was being designed
- You should state the statistical method of sample size computation and any required assumptions
- If no explicit power analysis was used, you should describe how you decided what sample (replicate) size (number) to use

Replicates

- You should report how often each experiment was performed
- You should include a definition of biological versus technical replication
- The data obtained should be provided and sufficient information should be provided to indicate the number of independent biological and/or technical replicates
- If you encountered any outliers, you should describe how these were handled
- Criteria for exclusion/inclusion of data should be clearly stated
- High-throughput sequence data should be uploaded before submission, with a private link for reviewers provided (these are available from both GEO and ArrayExpress)

Statistical reporting

- Statistical analysis methods should be described and justified
- Raw data should be presented in figures whenever informative to do so (typically when N per group is less than 10)
- For each experiment, you should identify the statistical tests used, exact values of N, definitions of center, methods of multiple test correction, and dispersion and precision measures (e.g., mean, median, SD, SEM, confidence intervals; and, for the major substantive results, a measure of effect size (e.g., Pearson's r, Cohen's d)
- Report exact p-values wherever possible alongside the summary statistics and 95% confidence intervals. These should be reported for all key questions and not only when the p-value is less than 0.05.

Group allocation

- Indicate how samples were allocated into experimental groups (in the case of clinical studies,

please specify allocation to treatment method); if randomization was used, please also state if restricted randomization was applied

- Indicate if masking was used during group allocation, data collection and/or data analysis
